# Disease management experiences of parents of children with ADHD: a qualitative directed content analysis

**DOI:** 10.3389/fpsyg.2026.1778650

**Published:** 2026-09-30

**Authors:** Yu Xia, Yanjiao Wang, Ying Tang, Xu Su

**Affiliations:** 1 Department of Psychiatry, The First Affiliated Hospital of Kunming Medical University, Kunming, China; 2 Department of Respiratory Medicine, Zhoushan Hospital of Traditional Chinese Medicine, Zhoushan, China; 3 Intensive Care Unit, The First Affiliated Hospital of Kunming Medical University, Kunming, China

**Keywords:** ABC-X model, ADHD, disease management, parents, qualitative research

## Abstract

**Background:**

The long-term management of children with ADHD primarily relies on parents. As primary caregivers, parents bear the main responsibility for their children’s treatment coordination, daily behavior guidance, and school collaboration. However, the subjective experiences of these parents in navigating ADHD management, including their challenges, coping strategies, and emotional responses, remain insufficiently explored.

**Methods:**

Face-to-face, semi-structured in-depth interviews were conducted with 17 parents of children with ADHD. These caregivers were recruited from the psychiatric outpatient department of a Grade A tertiary general hospital in Yunnan, China, via a purposive sampling method. Guided by the ABC-X Model of Family Stress and Adaptation as the theoretical framework, the interview data were analyzed using directed content analysis, with NVivo 12.0 software employed for data management and analysis.

**Results:**

The study identified four core categories corresponding to the ABC-X Model. (1) Multifaceted Challenges in Treatment Implementation: Medication Adherence Barriers, Time and Financial Pressures Faced. (2) Coping Resources: Positive Self-Regulation–Agentic Fatalism; Social Support: Gains and Gaps. (3) Differentiated Cognition of Disease Management: Evolution of ADHD Cognition, Perception and Cognitive Divergence in Disease Management Difficulty. (4) Duality of Management Results: Crisis Responses, Adaptive Outcomes. Other research findings included the impact of ADHD on siblings and social cognitive biases.

**Conclusion:**

The ABC-X Model is applicable to Chinese ADHD family management. The results of this study provide healthcare providers with a more detailed understanding of the disease management experiences of parents of children with ADHD.

## Introduction

The global prevalence of Attention-Deficit/Hyperactivity Disorder (ADHD) in children and adolescents stood at 8.0% (Ayano et al., 2024), while the corresponding prevalence was approximately 6.4% among school-aged children and adolescents in China (Liu et al., 2025). ADHD management is a long-term process involving medication, behavioral intervention, school adaptation guidance, and family environment adjustment (Peterson et al., 2024). Parents, as the primary implementers of home-based management and coordinators of multi-sector support (e.g., hospitals, schools), play a pivotal role in treatment adherence and outcome (Dvorsky et al., 2021; Evans and Evans, 1983). Effective caregiving can significantly reduce the recurrence of ADHD symptoms and improve children’s academic and social functioning (Kamimura-Nishimura et al., 2019). However, parents often experience overwhelming stress; studies indicate they are at high risk for emotional exhaustion, social isolation, and reduced quality of life, directly influencing child prognosis (Sarıkaya Erdil et al., 2023; Pardo-Salamanca et al., 2024). Existing studies on ADHD have mostly focused on children’s symptom improvement and medical intervention effects, while relatively few have explored parents’ subjective experiences of disease management, such as the stress they face, the resources they rely on, and how their cognitive attitudes shape management behaviors (Craig et al., 2020).

The ABC-X Model, proposed by Reuben Hill and further expanded by subsequent scholars, serves as a classic theoretical framework for analyzing family stress and adaptation (Hill, 1958). It comprises four core components: (1) A (Stressor event): An event or situation that disrupts family functioning; (2) B (Coping resources): Internal and external resources available to the family to cope with stress; (3) C (Cognitive appraisal): The family’s subjective interpretation and evaluation of the stressor; (4) X (Crisis outcome): The family’s adaptation or crisis response after encountering the stressor. This model has been widely applied in the study of family experiences with chronic diseases (e.g., dystrophinopathy, chronic kidney disease) (Frishman et al., 2017; Li et al., 2021), but its application to ADHD family management remains limited. This is particularly relevant in China’s “child-centered” parenting culture, where caregivers may hold unique cognitive biases and face distinct resource gaps (Lu et al., 2024).

Thus, this study employed the ABC-X Model to explore the disease management experiences of Chinese parents of children with ADHD, in order to help healthcare providers more accurately understand the needs of parents of children with ADHD during the disease management process.

## Methods

This study utilized directed content analysis guided by the ABC-X Model. This approach is suitable for research scenarios where existing theories can provide a predefined analytical framework, enabling systematic alignment between collected data and theoretical constructs to ensure analytical rigor (Hsieh and Shannon, 2005). All patients provided informed consent to participate in this study. Research carried out in this study is in compliance with the Helsinki Declaration. All research procedures have been approved by the Ethics Committee of the First Affiliated Hospital of Kunming Medical University (Ethics Approval No. 2024L271), and the study was conducted in full compliance with China’s ethical norms and relevant laws and regulations governing human biomedical research.

### Ethical considerations

Prior to conducting interviews, researchers provided all participants with a detailed explanation of the study’s purpose, methodology, data confidentiality measures (all information related to the participants is anonymized and kept confidential), and the right to withdraw from the study at any stage without reason or penalty. After participants fully understood the study and confirmed no questions remained, all participants signed a written informed consent form to voluntarily participate in the study. During the interview, if they showed negative emotional reactions, such as crying or sobbing, the researchers would pause the interview and provide emotional support.

### Study setting and participants

Participants were primary parents of children diagnosed with ADHD who attended the psychiatric outpatient department of a Grade A tertiary general hospital in Yunnan, China. This hospital provides medical services for patients from 16 prefectures and cities in Yunnan Province as well as surrounding provinces such as Sichuan and Guizhou. Purposive sampling was used for recruitment, which took place from November 2024 to February 2025. Throughout the recruitment period, sampling, interviewing, and data analysis proceeded concurrently. The research team continuously summarized and analyzed the demographic characteristics of enrolled caregivers and the clinical features of children with ADHD. To achieve maximum variation, we actively supplemented underrepresented subgroups based on the ongoing analysis of the emerging sample profile. Specifically, participants with diverse educational backgrounds, different economic levels, and varied caregiving durations were purposefully recruited. Meanwhile, children with different ADHD subtypes and disease durations were included to ensure the heterogeneity of the sample. This maximum variation was achieved within the study sample, and no matching was conducted against the actual demographic composition of ADHD families from the participating hospital or the local region. Following initial contact with parents who expressed interest in the study, the research team performed systematic screening. This included direct inquiries regarding the parents’ physical and mental health status and whether they were concurrently participating in other studies. Eligibility assessments were primarily based on parental self-reports, supplemented by the researchers’ evaluation of the parents’ ability to understand study information and maintain coherent communication. A total of 23 parents were initially assessed for eligibility. Among them, 4 declined to participate due to loss of interest, and 2 were excluded because they were unable to accommodate the study schedule. Finally, 17 parents were included in the study. This sampling strategy was adopted to ensure data diversity by capturing varied demographic profiles of caregivers (age, educational attainment, family structure, and household income) and variable clinical characteristics of children (ADHD subtypes and disease duration). The inclusion criteria were as follows: (1) primary parental caregivers of children aged ≤ 18 years with a formal ADHD diagnosis in accordance with the Diagnostic and Statistical Manual of Mental Disorders, Fifth Edition (DSM-5); (2) individuals responsible for the child’s daily disease management for a minimum of 3 months; (3) ability to communicate effectively in Mandarin or Yunnan dialect; (4) voluntary participation with written informed consent. Exclusion criteria included caregivers with severe mental health conditions (e.g., schizophrenia, major depressive disorder) that could compromise the authenticity and objectivity of self-reported data.

### Data collection

#### Interview guide development

The initial interview guide was developed based on a literature review and the ABC-X Model, covering four core dimensions of the model: (1) A (Stressor event); (2) B (Coping resources); (3) C (Cognitive appraisal); (4) X (Crisis outcome). Additionally, two caregivers of children with ADHD were selected for pilot interviews (they also signed the informed consent form), and the guide was refined based on their feedback to improve clarity and relevance, resulting in the final interview guide. The guide included 6 open-ended questions: 1. What experiences and emotions have you had in the process of caring for your child with ADHD? 2. What challenges have you encountered during the caregiving of your child with ADHD? 3. How do you currently perceive this condition (i.e., ADHD)? 4. Do you identify any key aspects that require attention when caring for children with ADHD? 5. Have you ever received external support (e.g., from family members, community organizations, or healthcare providers) in this caregiving process? 6. How do you cope with the challenges you have encountered while caring for your child with ADHD?

#### Interview implementation

All interviews were conducted by the interviewer (YX), who is a graduate student majoring in psychiatric nursing and has received specialized training in qualitative research and clinical knowledge of ADHD. Prior to the interviews, the interviewer (YX), working as an intern, spent 3 months in the research setting to familiarize herself with the study population. The interviews were held in a quiet and private consulting room in the outpatient department, with each session lasting 45 to 75 min. All interviews were audio-recorded with the participants’ consent. To reduce response bias, the interviews began with open-ended questions to build rapport (e.g., “Can you talk about the daily life after your child was diagnosed with ADHD?”), followed by targeted questions regarding each dimension of the ABC-X Model. Throughout the entire interview process, the researchers maintained a neutral stance and avoided leading questions. When participants provided brief answers, probing questions (e.g., “Can you elaborate further on this point?”) were used to deepen understanding, ensuring the collection of rich and in-depth qualitative data. During interviews, the interviewer (YX) briefly paraphrased participants’ key statements in real time to verify accurate understanding, ensuring the authenticity of the collected information.

#### Sample size determination

Consistent with qualitative research principles, the final sample size was determined by data saturation rather than a pre-specified number. Participant recruitment was discontinued iteratively when three consecutive interviews yielded no novel information, concepts, or thematic insights, ensuring sufficient data richness and analytical comprehensiveness. Sampling continued until data saturation was achieved. After 17 interviews, no new themes emerged during analysis, and the research team determined that data saturation had been reached, thus terminating recruitment (Francis et al., 2010).

#### Translation procedures

All interviews were conducted in Mandarin or Yunnan dialect and transcribed into standard written Chinese. All qualitative quotations were independently translated into English by two bilingual researchers with qualitative research experience. A standard forward-back translation procedure and bilingual cross-checks were performed to ensure translational accuracy and contextual consistency.

### Data analysis

All audio recordings were transcribed verbatim within 24 h after each interview, and all transcripts were cross-checked against the original audio recordings to ensure accuracy. Transcripts were imported into NVivo 12.0 software for coding and analysis. A directed content analysis was performed to deductively analyze the transcripts for dimensions of the theoretical model. Then, through iterative discussion, emergent categories were inductively explored (Hsieh and Shannon, 2005). Two researchers (YX and YT) with systematic training in qualitative methodology independently coded all transcripts. Specifically, the two coders completed the initial coding of the first interview transcript and formulated a standardized coding manual through repeated discussion and consensus negotiation to unify coding standards and terminological consistency. With the finalized coding manual, all remaining interview transcripts were subsequently coded independently by the two researchers in a unified manner. Data collection and data analysis were conducted concurrently to facilitate the identification of emerging themes. Any coding discrepancies were resolved through consultation with a senior researcher (YW), who has extensive experience in psychiatric nursing qualitative research. Given the limited visiting time of outpatient participants and to avoid imposing additional burden on caregivers, member checking was not implemented in this study.

### Trustworthiness and rigor

Trustworthiness and methodological rigor were strictly ensured in this study following four core dimensions: credibility, dependability, confirmability, and transferability (Lincoln and Guba, 1985).

For credibility, pilot interviews and flexible probing strategies were adopted to acquire in-depth original narratives. Concurrent data collection and analysis, together with maximum variation sampling, strengthened the authenticity and depth of qualitative findings.

Dependability was achieved through standardized research procedures, unified coding criteria, and independent dual coding by two researchers. All transcripts were carefully verified against original audio records to reduce procedural bias and guarantee analytical stability.

In terms of confirmability, all thematic analysis results were objectively summarized based on the original descriptions of the research participants. The entire research process, including participant screening, interview implementation, coding, and resolution of coding discrepancies, was clearly documented to avoid subjective interpretation and ensure the traceability of the analytical process.

Transferability was enhanced by detailed reporting of the research setting, participant demographics, and clinical characteristics of children with ADHD. Such contextual information enables other researchers to evaluate the applicability of the results in similar cultural and clinical environments.

## Results

### Participant characteristics

The participants consisted of 17 parents (10 mothers and 7 fathers), ranging in age from 35 to 46 years. Table 1 presents the demographic and clinical characteristics of the 17 caregivers and their children with ADHD. The main variables included parent–child relationship, parental age, educational level, number of children in the family, children’s gender and age, ADHD subtype, disease management duration, and household economic status.

**Table 1:** Demographic characteristics of parents and their children (*n* = 17).

Characteristics	Category	*n*
Parent age	≤40 years old	8
	41–45 years old	7
	≥46 years old	2
Parent education	Primary school	1
	Middle school	3
	High School	4
	College Degree	5
	Bachelor’s Degree	3
	Master’s Degree	1
Number of children	1	8
	2	9
Child gender	Boy	10
	Girl	7
Child age	7–10 years old	6
	11–14 years old	8
	≥15 years old	3
ADHD subtype	PI	11
	PHI	4
	C	2
Management duration	<1 year	1
	1–2 years (inclusive)	6
	2–4 years (inclusive)	6
	≥5 years	4
Economic status (annual)	Poor	5
	Average	7
	Good	4
	Excellent	1
Relationship with child	Mother	10
	Father	7

PI, Predominantly Inattentive presentation; PHI, Predominantly Hyperactive–Impulsive presentation; C, Combined presentation (per DSM-5 criteria). Economic status categories (annual household income): <20,000 CNY = Poor; 20,000–60,000 CNY = Average; 60,000–120,000 CNY = Good; >120,000 CNY = Excellent.

### Categories

#### Multifaceted challenges in treatment implementation

##### Medication adherence barriers

Medication adherence barriers are an important source of stress for parents of children with ADHD. Most parents say that when giving their children medication, they tell their children that they are taking vitamins instead of methylphenidate because they believe the children will resist taking the medication.

I tried telling my kid before that this was medicine to cure an illness, but as a result, he cried and shouted, “I’m not sick, I won’t take the medicine.” After that, it was particularly difficult to get him to take the medicine. Now, there’s no other way; I can only trick him into thinking it’s a vitamin. I only feel relieved when I see him take it obediently. Although I know lying is wrong, compared to him resisting taking the medicine and his condition being affected, there really is no other way (P13).

A subset of parents with full-time employment have admitted that, due to the time constraints and busyness of their work, they frequently have no alternative but to ask their children to carry medications to school independently for self-administration. However, children almost invariably fail to take their medications as scheduled. Confronted with this situation, these parents experience a strong sense of helplessness, as they have not yet identified a more appropriate and effective solution to address the issue for the time being.

Sometimes I’m in such a rush in the morning that I just let my kid take their medicine to school and take it there themselves. But eight out of ten times they forget. Every time I ask, they just say “I forgot” or “It slipped my mind.” I’m really at a loss and don’t know what to do… [a helpless laugh] (P15).

##### Time and financial pressures faced

Time-related and economic pressures also constitute key dimensions of stressors for parents during disease management. Balancing work responsibilities with caregiving obligations has emerged as a significant challenge. Parents frequently face the necessity of taking leave from work to attend medical appointments specifically required due to their children’s ADHD.

My wife and I can’t quit our jobs or leave early, we have to work. But we also need to take our child to the hospital, which makes time management impossible (P6).

My daughter missed so much school because of her ADHD that I had to take time off work to stay home with her (P3).

Long-term medication costs and additional rehabilitation interventions (e.g., sensory integration training) imposed significant financial pressure on families.

We’ve spent so much money on this disease. We had to stop sensory integration training because it was too expensive (P7).

We barely make ends meet. We paid over 3,000 RMB for a short sensory training course, but we couldn’t afford to continue. Even medication costs 600 RMB a month, I always wish there was a cheaper option (P11).

#### Coping resources

##### Positive self-regulation–agentic fatalism

Positive self-regulation is one of the important aspects of the resources that individuals can call upon internally. In this study, most parents acknowledged ADHD as a “fate-related reality” through positive self-regulation, but still persisted in their efforts to improve.

My mother always says, ‘This is fate, ‘yet this does not mean I can stand by and do nothing. I take my child for regular follow-up appointments, remind him to take medication every day, and also handle other related matters. Even though I am exhausted, I must keep trying. If things do not improve in the end, I will accept this outcome, but I will never give up at this moment (P12).

##### Social support: gains and gaps

Family members’ support constitutes a crucial component of resources for parents of children with ADHD during the disease management process. The mutual sharing of ADHD-related care tasks among family members not only helps parents better address challenges encountered in disease management but also strengthens family cohesion in some cases.

On workdays, my parents-in-law assist us in taking care of our child, and we divide responsibilities for the child’s daily care. Compared to the past when only my husband and I were responsible for the child, I am truly grateful for my parents-in-law’s understanding, they have prevented us from being overwhelmed, and the entire family has become more harmonious (P16).

However, some parents reported limited understanding and collaboration from other family members, which often led to conflict.

*My husband and sister do not think our child needs medication; they say ADHD is not a big deal*. *No one supports me, and we have fought so many times (P1)*.

Peer support from other parents of children with ADHD is highly valued. Parents use social media groups to share tips, and reduce feelings of isolation.

In the WeChat group for parents of children with ADHD, we share various disease management strategies, such as using a timer to break down homework tasks for the child and rewarding the child with stickers when they can stay focused. Communicating with individuals who understand our experiences, rather than those who pass judgment, is particularly meaningful (P8).

Community support for parents of children with ADHD was notably lacking.

Community health centers occasionally held parenting seminars before, but none of them focused on ADHD. I suggested to community staff whether they could invite a doctor to give lectures on how to manage children with this condition, yet they responded, “The demand is too low, so it’s difficult to organize.” It feels like parents of children with ADHD are barely attended to at all. (P5)

Healthcare support for ADHD management, particularly in terms of follow-up care, was inadequate.

We pick up medication approximately once a month, and there is no one available to consult via phone if problems arise in between. (P2)

Parents expressed urgent needs for support from multiple social sectors. They requested more targeted education from healthcare providers to help identify reliable ADHD-related resources.

We need yours [healthcare staff] to teach us how to identify good resources during training—this kind of science popularization is really needed (P5).

#### Differentiated cognition of disease management

##### Evolution of ADHD cognition

Evolution of ADHD Cognition constitutes a key dimension of cognitive appraisal. Most parents pointed out that they initially misunderstood ADHD, attributed their children’s behavioral problems to their own inadequacy, and even regarded themselves as incompetent parents.

When my 8-year-old son was in primary school, his teacher frequently reported that he “could not sit still” and “disrupted class activities.” In response, I would yell at him upon returning home, and even resorted to spanking him on several occasions. I kept questioning myself: “Why can’t he behave like other children? Am I being too indulgent with him?” At that time, I had no awareness that his behaviors were symptoms of a neurodevelopmental disorder. Instead, I solely attributed the issue to my own inadequacy and perceived myself as a failing mother (P4).

A subset of parents admitted that remarks from people around them often deepened their misunderstandings, leading them to mistakenly attribute ADHD symptoms to innate traits of the child rather than recognizing them as a medical issue.

I talked to a neighbor whose child was also quite ‘hyperactive’. She said, ‘Oh, my son grew out of that. It’s just a phase of childhood mischief.’ So, I waited. I thought, ‘Maybe one day he’ll calm down on his own.’ However, his behaviors only worsened, he struggled to concentrate and frequently got into conflicts with other children. Later, I felt foolish: Why did I trust my neighbor instead of the doctor? But back then, she was the only ‘expert’ I had access to (P14).

##### Perception and cognitive divergence in disease

Perception and Cognitive Divergence in Disease is another important aspect of cognitive appraisal. Parents with a negative appraisal attitude feel overwhelmed by the persistent demands of ADHD management.

I have to supervise him to do homework all the time every day; if I take my eyes off him for just a moment, he starts playing with erasers or biting his fingers. I remind him to correct this behavior, but he goes back to doing it again within two minutes. I joined two [online groups], but both turned out to be full of people selling health supplements. I really don’t know what to do. Sometimes when I look at him, I feel extremely exhausted, as if no matter how hard I try, it makes no difference (P12).

Parents with a positive appraisal attitude perceive ADHD management as a solvable challenge. They emphasize agency and hope, while reframing “struggles” as “steps toward progress.”

I used to think that ADHD meant my son would ‘never keep up with his peers,’ but now I hold a different perspective. He has difficulty sitting still, yet he possesses a remarkable talent for building things, so we enrolled him in a Lego club. When it comes to doing homework, we break the tasks into 10-minute segments instead of tackling them in one full hour. Last night, he completed his math workbook without getting distracted even once! While this process is not easy, every small success reminds me that we are not fighting a losing battle; we are simply finding our own pace (P16).

#### Duality of management results

##### Crisis responses

Parents’ coping strategies directly affected the effectiveness of disease management, leading to two distinct outcomes in terms of symptom control and child well-being. Parents’ disease management experiences yield two types of outcomes: either crisis responses or adaptive outcomes, which depend on the interaction between stressors, resources, and cognitive appraisal. Families with high stress, limited resources, and negative cognitive appraisal have experienced crises such as caregiver burnout and parent–child conflicts.

As he grows older, we often end up in arguments followed by cold silence. I don’t even want to take the initiative to reconcile anymore, because I’m truly exhausted! I take care of him day in and day out, but looking after him isn’t my only responsibility. I still have to go to work and do household chores, and my own health has declined because of his illness (P9)!

##### Adaptive outcomes

When coping resources are sufficient and cognitive appraisal is positive, parents achieve adaptive outcomes, including the development of standardized management strategies and improved parent–child communication.

During my child’s follow-up visits, I have met many other parents of children with similar conditions, from whom I have learned practical experiences in caring for my child. As a full-time mother, I have created a daily schedule for my child to help her develop healthy living habits, and I accompany her to engage in moderate physical activities every day. Currently, our daily life has become more organized and stable (P17).

#### Beyond the Core ABC-X framework

##### Impact on siblings

Beyond the core themes of the ABC-X framework, parents described a distinct set of experiences related to unaffected siblings. These dynamics, ranging from emotional neglect to unexpected positive adaptations, extend the model to include broader family-level impacts. When parents’ energy is mostly directed towards children with ADHD, the psychological needs and daily companionship of non-affected children are easily overlooked.

My daughter says, ‘after my brother got sick you never help me with homework anymore’ (P14).

When families need to invest energy in caring for children with ADHD, some older children will voluntarily participate in the care process, support their parents and siblings through their actions, and demonstrate empathy and a sense of responsibility beyond their age.

My 12-year-old daughter helps her 7-year-old sister remember her meds. She says, ‘she can’t help her ADHD, so I’ll help her.’ It makes me proud (P3).

##### Society’s perception bias towards ADHD

Beyond the core themes aligned with the ABC-X framework, caregivers described distinct experiences related to societal perceptions of ADHD. Parents say that many people around them still regard ADHD as “just being naughty and mischievous” or “lacking discipline” rather than a neurodevelopmental disorder, and some even think it is a “non-existent disease.” This cognitive bias stems from insufficient public education. Society often pays more attention to popularizing awareness of severe mental illnesses such as depression and schizophrenia, leaving ADHD in a cognitive blind spot.

After my child was diagnosed with ADHD, I discussed this matter with many people around me. Most people have little understanding of ADHD and think it's not a real illness. There is too little public education about ADHD in our society. Perhaps people pay more attention to severe mental illnesses, such as depression or schizophrenia (P1).

Many parents admit that, influenced by stereotypes, most members of the public default to perceiving ADHD as a “male-only” condition. They not only overlook the symptomatic manifestations of ADHD in girls but also tend to question and deny the diagnosis, even when girls have received a professional confirmation of the disorder.

When my cousin learned that my daughter had been diagnosed with ADHD, she expressed great surprise, saying, “That can’t be right, can it? Isn’t ADHD something only boys get? Your daughter seems quietly, she’s just a bit distracted sometimes. Don’t make a mountain out of a molehill.” Her words made me feel quite upset, as I felt no one could truly understand our situation (P8).

## Discussion

This study explored the disease management experiences of parents of 17 children with ADHD in Kunming. We found that the disease management experiences of parents of children with ADHD are not solely determined by objective barriers during the implementation of treatment, but rather by the interaction between coping capabilities and differentiated perceptions of disease management, which either buffer or amplify stress, consistent with the core premise of the ABC-X Model.

The parents in this study faced multiple stressors, mainly including three aspects: children’s medication adherence barriers, as well as the time and economic pressures brought about by long-term treatment. These aspects align with the A (stressor events) dimension of the ABC-X Model. Such pressures have prompted parents to actively seek internal and external coping resources, including intra-family collaboration and external medical or community support. Therefore, identifying the types and intensity of stressful events is the primary step to understanding family coping behaviors. Previous studies also support this view: among families of children with chronic illnesses, the more complex the stressors they face, the heavier the coping burden on parents and the higher their reliance on external resources (Yadav, 2024; McLoone et al., 2022).

In our interviews, parents frequently emphasized the crucial role of coping resources in alleviating stress, which is consistent with the B (resources) dimension of the ABC-X Model. Their coping resources were characterized by “strong internal regulation yet weak external support”: when internal resources (such as self-regulation capabilities) and external resources (such as spousal support, online peer assistance, and community services) were sufficient, the impact of stress was significantly mitigated (Hill, 1958). In contrast, parents with insufficient resources, such as those lacking spousal understanding or specialized community support, often fell into a dilemma of “wanting to manage but being unable to do so,” and even delayed treatment due to the chaos of online information. This is consistent with the findings of Li et al. (2021) in their study on families of children with chronic kidney disease, where resource gaps directly amplify the impact of stress and reduce family coping efficiency. Similarly, Ching’oma et al. (2022) noted in their study on ADHD families in Tanzania that the lack of community support is a key factor exacerbating parents’ caregiving burden. Additionally, this study found that parents regarded “intra-family collaboration” as a critical supportive resource in the process of disease management. This may be attributed to the influence of the “family-oriented” culture on Chinese parents, leading to a high reliance on internal family collaboration. Relevant studies have reported similar phenomena: a phenomenological study revealed that grandmothers, as important participants in family collaboration, take the initiative to share practical caregiving work, and such collaboration becomes a key resource for parents to cope with the disease (Huang et al., 2020).

Within the coping-resource dimension, a distinctive coping pattern emerged from our data: agentic fatalism. Parents accept the chronic nature of ADHD yet persist in active caregiving interventions. This pattern can be interpreted using the analytical framework of assimilative and accommodative coping. Assimilative coping refers to efforts to alter external circumstances, whereas accommodative coping involves adjusting one’s own cognitions to accept unchangeable realities (Brandtstädter and Renner, 1990). Agentic fatalism represents a hybrid coping style: parents acknowledge the chronic and incurable nature of ADHD (an accommodative stance) but still take agentic actions, including proactively screening authoritative medical information, mobilizing family members to share caregiving responsibilities, and gradually establishing a standardized disease-management mechanism. This pattern is not passive resignation; instead, it reflects the dynamic interplay between fatalistic acceptance of illness constraints and purposeful, proactive action. As an analytical category derived from participants’ real-life experiences, this construct extends existing literature on fatalism and agency among family caregivers (Park, 2010; Purc-Stephenson and Edwards, 2024).

In addition, our study indicates that parents’ cognitive appraisal (C) of ADHD plays a crucial mediating role between stress (A) and resources (B), which is consistent with the logic of the ABC-X Model that “cognition determines the efficiency of resource utilization.” Parents who perceive ADHD as a “manageable neurodevelopmental disorder” (positive cognitive appraisal) will take the initiative to mobilize resources, such as joining online parent groups to acquire practical skills and strictly following doctors’ recommendations for medication treatment. In contrast, parents with negative cognitive appraisal, who mistakenly categorize ADHD as “children’s misbehavior” or “educational failure,” may overlook scientific resources, even resist treatment, and instead rely on “folk remedies” or “wait for spontaneous recovery.” Previous studies have also corroborated this perspective, namely that parents’ attribution of ADHD directly influences their treatment decisions (Peasgood et al., 2016; Coletti et al., 2012).

Our study demonstrates that crisis outcomes (X) vary depending on the interaction of the aforementioned three dimensions. Families characterized by high stress, limited resources, and negative cognitive appraisals have experienced crises such as caregiver burnout and parent–child conflicts. In contrast, those with adequate resources—including family support, peer groups, and positive appraisals—have achieved adaptive outcomes, such as improved daily routines and parent–child communication. This supports the proposition of the ABC-X Model that crises are not inevitable but rather depend on how families mobilize resources and interpret stress (Hill, 1958).

We also identified two unique aspects of the disease management experiences of parents of children with ADHD that are not encompassed in the ABC-X Model.

The first unique aspect is “the impact on siblings.” The ABC-X Model focuses on the stress, resources, and cognition of parents as primary caregivers. However, parents commonly mentioned in the interviews that the siblings of children with ADHD are not only affected by the disease management process but may also take an active part in it, specifically manifesting in two types of situations: “emotional loss” and “responsibility assumption.” “Emotional loss” mostly stems from the reduction in parental companionship time, which makes siblings generate a sense of being neglected, and may further arouse resistance toward their ADHD siblings, ultimately exacerbating internal family conflicts. In contrast, “responsibility assumption” is reflected in some elder siblings proactively undertaking caregiving tasks such as reminding the ADHD child to take medication and assisting with regular daily routines. This role as “young caregivers” not only effectively alleviates the parents’ caregiving burden but also promotes the formation of a “mutual support network” within the family, and may even provide emotional comfort to the exhausted parents. This is consistent with the findings of existing studies: although siblings without ADHD may experience a sense of being overlooked due to perceiving differences in parental treatment, they often develop ways to interact with their ADHD siblings and provide support to their parents within their capabilities (King et al., 2016; Steinhausen et al., 2012). Second, our study found that Chinese society may have certain cognitive blind spots regarding ADHD. On one hand, there exists the stereotype that “ADHD only affects males,” resulting in the easy neglect of symptoms in female patients, and their parents are more likely to face doubts when seeking diagnosis and support. On the other hand, there is a prejudice that “ADHD is not a real disease”; some members of the public simply attribute symptoms such as inattention, hyperactivity, and impulsivity in affected children to “misbehavior” or “insufficient discipline.” This leaves parents frequently encountering misunderstandings and pressures in the process of disclosing the disease, engaging in social interactions, and communicating with schools. A large-scale study revealed that ADHD in girls may be underdiagnosed, possibly because the symptoms of ADHD in girls are not as “typical” as those in boys (Madsen et al., 2018; Bölte et al., 2023; Chandler, 2010). Existing studies have supported this perspective: the public may perceive children with ADHD as merely “naughty” or “misbehaving” rather than having a neurodevelopmental disorder. Such stigmatization may lead families to be reluctant to seek professional help, delaying diagnosis and intervention, which in turn affects the children’s treatment adherence and overall well-being (Pant, 2025; Yusnia and Kurniadewi, 2025).

In addition, this study also observed that the primary motivation for almost all parents to seek medical treatment initially was their children’s “decline in academic performance.” Against the social backdrop of “educational involution,” which refers to intensified academic competition, pervasive parental anxiety, and diminishing marginal returns on educational investment in China, academic performance is regarded as a crucial benchmark for children’s development (He and Liu, 2025). When ADHD leads to homework procrastination, low classroom efficiency, and a drop in academic grades, parents first experience anxiety about “their children falling behind in academic competition” rather than paying attention to the children’s potential neurodevelopmental issues (Jandre et al., 2024). Such a medical-seeking motivation centered on academic performance not only reflects the superimposition of current academic competition pressure and parental educational anxiety in China but may also result in excessive focus on academics while neglecting emotional and social adaptation needs in subsequent management. Therefore, when supporting ADHD families, it is necessary to guide parents to balance their academic expectations with their children’s holistic development. While working to improve attention, healthcare providers should also attend to children’s psychological and social health, avoiding deviation from the core goal of disease management due to a single focus on academic performance.

The findings of this study will inform healthcare providers working with children with ADHD. These results offer valuable insights into the experiences of parents of children with ADHD, helping healthcare providers understand the parents’ disease management journeys. For parents with insufficient resources, healthcare providers can proactively inquire about their difficulties in time allocation and the extent of their economic burden, provide timely access to the interpretation of medical insurance reimbursement policies, and alleviate their resource scarcity predicament. For cognitive biases, concise and accessible science popularization brochures can be developed to clarify the atypical symptoms of ADHD in girls, and parent–child communication templates for “scientifically interpreting ADHD” can be provided to correct parents’ management misconception of “valuing academic performance above all else” and guide them to focus on their children’s emotional regulation and social adaptation. In addition, consideration may be given to incorporating the adaptation of siblings into disease management interventions to help construct a sibling mutual support network. This will make medical support more aligned with the actual needs of families and enhance the effectiveness of ADHD disease management.

## Limitations

This study has several limitations. Participants were recruited from one Grade A tertiary general hospital in Yunnan Province, and the research subjects were limited to the biological parents of children with ADHD, excluding other potential caregivers such as grandparents. The singularity of the regional scope and caregiver identity may restrict the applicability of the research results. Furthermore, maximum-variation purposive sampling was used in this study, and heterogeneity was only ensured within the study sample without matching the real-world demographic profile of ADHD families at the study hospital or local area. This may further limit the generalizability of the present findings. Additionally, the study adopted a qualitative interview study design, only collecting static data during the period from 2024 to 2025, which failed to effectively track the dynamic changes in caregivers’ stress levels, accessible resources, and disease cognition throughout the long-term disease management process, thus affecting the comprehensive understanding of the research phenomenon. Furthermore, all information regarding the impact of children with ADHD on their siblings was solely based on parental reports, and the subjective experiences of the siblings themselves were not collected in this study. In addition, cultural factors should be noted when interpreting these findings. Under the influence of traditional family values, Chinese families commonly adopt child-centered parenting and emphasize internal family collaboration in healthcare and daily rearing. This cultural backdrop shapes caregivers’ perceptions and ways of coping with chronic childhood conditions. Compared with individualistic societies that rely more on social and institutional support, family-based participation and care engagement are more prominent in this context. Therefore, while the core caregiving challenges may be universal, the specific manifestations (e.g., caregiving burden allocation and help-seeking patterns) and family coping models observed in this study might vary across different cultural environments. Correspondingly, caution should be exercised when generalizing these findings to individualistic cultural contexts.

Future research can be advanced from the following three aspects: firstly, conduct multi-center longitudinal studies, expand the regional coverage of samples and the types of caregivers, and explore the correlation between different disease stages and family management experiences through 1–3 years of continuous follow-up; secondly, pay attention to the potential impact of children with ADHD on the mental health of their siblings to further improve the research at the family system level; thirdly, adopt a mixed-methods research approach, combining quantitative surveys with in-depth family interviews to comprehensively present the mechanisms of action of family interaction patterns on disease management experiences.

## Conclusion

This qualitative study based on the ABC-X Model clarifies that the disease management experiences of parents of children with ADHD are shaped by multiple pressures in treatment implementation, internal and external family coping resources, differentiated cognition of disease management, and the interplay of these factors. The research findings not only further verify the applicability of the ABC-X Model in the study of family stress and adaptation among children with chronic neurodevelopmental disorders but also expand the multi-dimensional understanding of ADHD family management, revealing two unique dimensions not covered by the model: the impact on siblings and the social cognitive biases towards ADHD. This study provides important references for healthcare professionals to formulate targeted support strategies, which is conducive to optimizing the clinical practice and social support system of ADHD disease management and effectively meeting the actual needs of parents of children with ADHD.

## Data Availability

The raw data supporting the conclusions of this article will be made available by the authors, without undue reservation.

## References

[ref1] Ayano G. Tsegay L. Gizachew Y. Demelash S. Alati R. (2024). The prevalence of attention deficit hyperactivity disorder in children and adolescents: an umbrella review of global evidence. Eur. Psychiatry 67:S76. doi: 10.1192/j.eurpsy.2024.203, 37495084

[ref2] Bölte S. Neufeld J. Marschik P. B. Williams Z. J. Gallagher L. Lai M. C. (2023). Sex and gender in neurodevelopmental conditions. Nat. Rev. Neurol. 19, 136–159. doi: 10.1038/s41582-023-00774-6, 36747038 PMC10154737

[ref3] Brandtstädter J. Renner G. (1990). Tenacious goal pursuit and flexible goal adjustment: explication and age-related analysis of assimilative and accommodative strategies of coping. Psychol. Aging 5, 58–67. doi: 10.1037/0882-7974.5.1.58, 2317302

[ref4] Chandler C. (2010). The Science of ADHD: A guide for Parents and Professionals. Chichester: Wiley Blackwell.

[ref5] Ching’oma C. D. Mkoka D. A. Ambikile J. S. Iseselo M. K. (2022). Experiences and challenges of parents caring for children with attention-deficit hyperactivity disorder: a qualitative study in Dar es salaam, Tanzania. PLoS One 17:e0267773. doi: 10.1371/journal.pone.0267773, 35921363 PMC9348639

[ref6] Coletti D. J. Pappadopulos E. Katsiotas N. J. Berest A. Jensen P. S. Kafantaris V. (2012). Parent perspectives on the decision to initiate medication treatment of attention-deficit/hyperactivity disorder. J. Child Adolesc. Psychopharmacol. 22, 226–237. doi: 10.1089/cap.2011.0090, 22537185 PMC3374352

[ref7] Craig F. Savino R. Fanizza I. Lucarelli E. Russo L. Trabacca A. (2020). A systematic review of coping strategies in parents of children with attention deficit hyperactivity disorder (ADHD). Res. Dev. Disabil. 98:103571. doi: 10.1016/j.ridd.2020.103571, 31931455

[ref8] Dvorsky M. R. Friedman L. M. Spiess M. Pfiffner L. J. (2021). Patterns of parental adherence and the association to child and parenting outcomes following a multicomponent school-home intervention for youth with ADHD. Behav. Ther. 52, 745–760. doi: 10.1016/j.beth.2020.09.007, 33990247 PMC8124088

[ref9] Evans W. H. Evans S. S. (1983). Using parents in behavior management. Acad. Ther. 19, 37–41. doi: 10.1177/105345128301900106

[ref10] Francis J. J. Johnston M. Robertson C. Glidewell L. Entwistle V. Eccles M. P. . (2010). What is an adequate sample size? Operationalising data saturation for theory-based interview studies. Psychol. Health 25, 1229–1245. doi: 10.1080/08870440903194015, 20204937

[ref11] Frishman N. Conway K. C. Andrews J. Oleson J. Mathews K. Ciafaloni E. . (2017). Perceived quality of life among caregivers of children with a childhood-onset dystrophinopathy: a double ABCX model of caregiver stressors and perceived resources. Health Qual. Life Outcomes 15:33. doi: 10.1186/s12955-017-0612-1, 28187773 PMC5303295

[ref12] He Y. Liu X. (2025). Development and validation of academic involution scale for college students. Sci. Rep. 15:20876. doi: 10.1038/s41598-025-04850-3, 40592983 PMC12218362

[ref13] Hill R. (1958). Generic features of families under stress. Soc. Casework 39, 139–150. doi: 10.1177/1044389458039002-318, 42737707

[ref14] Hsieh H.-F. Shannon S. E. (2005). Three approaches to qualitative content analysis. Qual. Health Res. 15, 1277–1288. doi: 10.1177/104973230527668 16204405

[ref15] Huang Y.-P. Wang S.-Y. Kellett U. Chen C.-H. (2020). Shame, suffering, and believing in the family: the experiences of grandmothers of a grandchild with a developmental delay or disability in the context of Chinese culture. J. Fam. Nurs. 26, 52–64. doi: 10.1177/1074840719895264, 31910721

[ref16] Jandre C. R. S. Villaça A. A. Balbino M. S. de Miranda D. M. Nobre C. N. (2024). Personas and socially aware Design in the Characterization of children and adolescents with attention-deficit/hyperactivity disorder: a technological and social approach. Univ. Access Inf. Soc. 24, 1509–1526. doi: 10.1007/s10209-024-01156-5, 30311153

[ref17] Kamimura-Nishimura K. I. Brinkman W. B. Froehlich T. E. (2019). Strategies for improving ADHD medication adherence. Curr. Psychiatr. Ther. 18, 25–38, 33867872 PMC8049396

[ref18] King K. Alexander D. Seabi J. (2016). Siblings’ perceptions of their ADHD-diagnosed sibling’s impact on the family system. Int. J. Environ. Res. Public Health 13:910. doi: 10.3390/ijerph13090910, 27649212 PMC5036743

[ref19] Li K. Liu T. Wang X. Niu Y. Sun Y. Yang X. (2021). Current status and influencing factors of family resilience in children with chronic kidney disease. Chinese journal of. Nurs. Sci. 36, 29–32. doi: 10.3870/j.issn.1001-4152.2021.17.029

[ref20] Lincoln Y. S. Guba E. G. (1985). Naturalistic Inquiry. Thousand Oaks: SAGE, 289–331.

[ref21] Liu J. Jiang Z. Li F. Zheng Y. Cui Y. Xu H. . (2025). Prevalence and comorbidity of attention deficit hyperactivity disorder in Chinese school-attending students aged 6-16: a national survey. Ann. General Psychiatry 24:23. doi: 10.1186/s12991-025-00558-w, 40223102 PMC11995565

[ref22] Lu H. J. Zhu N. Chen B. B. Chang L. (2024). Cultural values, parenting, and child adjustment in China. Int. J. Psychol. 59, 512–521. doi: 10.1002/ijop.13100, 38097526 PMC11176261

[ref23] Madsen K. B. Ravn M. H. Arnfred J. Olsen J. Rask C. U. Obel C. (2018). Characteristics of undiagnosed children with parent-reported ADHD behaviour. Eur. Child Adolesc. Psychiatry 27, 149–158. doi: 10.1007/s00787-017-1029-4, 28733915

[ref24] McLoone J. Wakefield C. E. Marshall G. M. Pierce K. Jaffe A. Bye A. . (2022). It’s made a really hard situation even more difficult: the impact of COVID-19 on families of children with chronic illness. PLoS One 17:e0273622. doi: 10.1371/journal.pone.0273622, 36048846 PMC9436103

[ref25] Pant L. (2025). “ADHD isn’t real” – addressing ADHD stigma through public policy and social marketing strategy. Soc. Mark. Q. 31, 384–407. doi: 10.1177/15245004251376014

[ref26] Pardo-Salamanca A. Rosa-Martínez E. Gómez S. Santamarina-Siurana C. Berenguer C. (2024). Parenting stress in autistic and ADHD children: implications of social support and child characteristics. J. Autism Dev. Disord. 55, 2284–2293. doi: 10.1007/s10803-024-06377-4, 38700780 PMC12167323

[ref27] Park C. L. (2010). Making sense of the meaning literature: an integrative review of meaning making and its effects on adjustment to stressful life events. Psychol. Bull. 136, 257–301. doi: 10.1037/a0018301, 20192563

[ref28] Peasgood T. Bhardwaj A. Biggs K. Brazier J. E. Coghill D. Cooper C. L. . (2016). The impact of ADHD on the health and well-being of ADHD children and their siblings. Eur. Child Adolesc. Psychiatry 25, 1217–1231. doi: 10.1007/s00787-016-0841-6, 27037707 PMC5083759

[ref29] Peterson B. S. Trampush J. Maglione M. Bolshakova M. Rozelle M. Miles J. . (2024). Treatments for ADHD in children and adolescents: a systematic review. Pediatrics 153:e2024065787. doi: 10.1542/peds.2024-065787, 38523592

[ref30] Purc-Stephenson R. J. Edwards R. (2024). Finding meaning in chronic illness and its relationship to psychological well-being: a mixed-methods study. PLOS Ment Health 1:e0000121. Published 2024 Sep 5. doi: 10.1371/journal.pmen.0000121, 41661854 PMC12798621

[ref31] Sarıkaya Erdil S. Yeni Elbay R. Karadere E. Görmez A. (2023). Parental stress in the parents of the children diagnosed with ADHD. Child Family Behav. Ther. 46, 27–41. doi: 10.1080/07317107.2023.2240311, 37339054

[ref32] Steinhausen H.-C. Zülli-Weilenmann N. Brandeis D. Müller U. C. Valko L. Drechsler R. (2012). The behavioural profile of children with attention-deficit/hyperactivity disorder and of their siblings. Eur. Child Adolesc. Psychiatry 21, 157–164. doi: 10.1007/s00787-012-0249-x, 22297660

[ref33] Yadav P. K. (2024). The impact of pediatric chronic illness on family dynamics and parental health: challenges and coping mechanisms. IJFMR 6:22732. doi: 10.36948/ijfmr.2024.v06i03.22732, 40520800

[ref34] Yusnia V. Kurniadewi E. (2025). Coping strategies of parents facing social stigma for having children with ADHD. Education 5, 87–105. doi: 10.51903/cfevs672

